# Migrating pleural plaque in a patient with asbestos induced pleural disease: a case report

**DOI:** 10.1186/s12995-017-0171-8

**Published:** 2017-08-24

**Authors:** Christian Eisenhawer, Michael K. Felten, Thomas Hager, Mikhail Gronostayskiy, Philipp Bruners, Andrea Tannapfel, Thomas Kraus

**Affiliations:** 1 Institute of Occupational and Social Medicine, RWTH Aachen University, Pauwelsstraße 30, 52074 Aachen, Germany; 2 Institute of Pathology, University Hospital of Essen, University of Duisburg-Essen, Hufelandstraße 55, 45147 Essen, Germany; 3 Division of Thoracic Surgery, West German Lung Center, Ruhrlandklinik, University Hospital Essen, Tüschener Weg 40, 45239 Essen, Germany; 4 Department of Diagnostic Radiology, RWTH Aachen University, Pauwelsstraße 30, 52074 Aachen, Germany; 5 Department of Pathology, Ruhr-University Bochum, Bürkle de la Camp-Platz 1, 44789 Bochum, Germany

**Keywords:** Asbestos, Plaques, CT-scan, Surveillance, Tumour

## Abstract

**Background:**

Health surveillance of formerly asbestos exposed individuals focus on early detection of asbestos related diseases, such as lung fibrosis (asbestosis), pleural plaques, mesothelioma and lung cancer in particular. One main concern is the early and clear identification of lesions with a high risk of malignant changes and their undelayed clinical work-up. False positive results may lead to unnecessary and often painful diagnostic interventions, which create high costs when applied to a large cohort and also may discredit the whole program. We describe an unusual presentation of a common lesion among asbestos exposed individuals, which has to our knowledge never been described before. Being aware of this pathological pathway may prevent inadequate clinical decisions with disadvantages for the patient. Underlying implications regarding health surveillance and the reading of CT-scans of the thorax are important for the management of formerly asbestos exposed individuals.

**Case presentation:**

During follow-up of an asbestos exposed 72 year old former power plant worker with known pleural changes, a nodule located next to the left costophrenic angle was newly discovered on CT-scan. As the previous scan 1 year before did not show any changes in that area, a fast growing tumour was suspected and an immediate biopsy performed. The tissue showed the characteristics of a pleural plaque with no signs of malignancy. After carefully reviewing all previous radiographs a rounded opacity attached to the mediastinal pleura close to the oesophagus and slightly cranial to the position of the removed nodule could be discerned. That nodule had increased in size over several years and was no longer visible on the latest scan. It appeared that the originally slow growing plaque had migrated to the costophrenic angle some time before it was discovered in the latest scan thus imposing as a fast growing tumour.

**Conclusions:**

We concluded that asbestos related pleural plaques can under special circumstances get separated from the pleura and migrate to another position in the pleural cavity. The case provides new insights in the development and properties of pleural lesions and may offer new options for the management of formerly asbestos exposed patients.

## Background

In formerly asbestos exposed individuals non-malignant changes of the pleura are usually developing over years in the form of plaques or diffuse pleural thickening, which do not require specific clinical work-up or intervention. As recommended in the Helsinki criteria for diagnosis and attribution for asbestos related diseases atypical changes on radiography related to the pleura call for immediate diagnostic work-up with biopsy to rule out early malignant mesothelioma and other malignancies [1–4]. While low-dose CT-scans are currently the most effective method of detecting asbestos related diseases, conventional chest X-ray and possibly ultrasonography of the chest are alternative techniques particularly suitable for low-risk groups [5].

In our surveillance program of formerly highly asbestos exposed employees with a diagnosis of non-malignant asbestos related disease of lung or pleura are invited for medical examinations annually or every other year. These examinations include a physical status, lung function testing and a CT-scan of the thorax. For CT examination a standard lowdose MDCT protocol is used (SOMATOM Sensation 16, Siemens Medical Solutions, Forchheim, Germany): 120 kV, individuals weighing less than 80 kg with 10mAseff/individuals weighing 80 kg and more with 20mAseff, 16 × 0.75 mm collimation, a rotation time of 0.5 s, and a table feed/rotation of 18 mm. The CT-scans are routinely read by a radiologist and an experienced occupational health specialist. In cases of disagreement an expert opinion can be obtained from an external specialist with particular experience in the field of asbestos related radiological changes. The examinations are mainly intended for early detection of mesothelioma, lung cancer and restrictive lung disease caused by proceeding lung fibrosis or pleural plaques. Such a diagnosis would entitle the patient for immediate clinical treatment, rehabilitation and possibly compensation payments by the Statutory Accident Insurance System. In addition to the medical diagnosis, proof of a significant cumulative asbestos exposure of 25 fibre-years or more is an important precondition for compensation. The early diagnosis of plaques and their differentiation from other pathological changes can be complicated and even “typical” results misleading [6–8].

## Case presentation

**Fig. 1:**
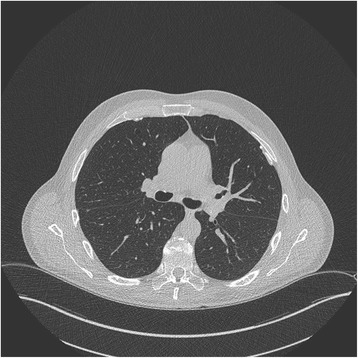
CT-scan of the thorax of 2014 with partially calcified plaque

In the year 2014 a 72 year old formerly asbestos exposed power plant worker (welder, fitter) presented for routine medical examination as participant of the surveillance program for patients with non-malignant asbestos related diseases. He had been registered for the program years earlier when bilateral partially calcified pleural plaques had been discovered (Fig. 1).

In comparison to the previous examination in 2013 the asbestos related changes showed no signs of progression. The patient could not recall any significant symptoms especially shortness of breath, coughing with or without sputum or chest pain. In the years 1959 to 1960 he had smoked about 10 cigarettes per day. Other than symptomatic treatment for prostatic hyperplasia, he took no oral medication. The physical status, including auscultation of heart and lungs, was unremarkable and consistent with his age. The bodyplethysmographic lung function test showed no signs of restrictive or obstructive lung disease, and no indication of emphysema. The results of capillary blood gas analysis and single breath carbon monoxide diffusion before and during standardised cycle ergometry (100 W for 4 min) showed no signs of impaired ventilation.

**Fig. 2:**
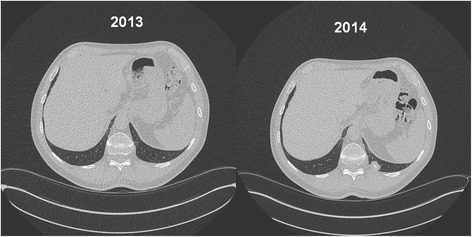
CT-scans of the thorax (*left*: 2013, *right*: 2014) showing identical levels of the costophrenic sinus, with a newly emerged large, smooth nodule paravertebral attached to the *left* chest wall (dimensions 21 × 13 mm, central calcification, no contrast material)

The direct comparison of CT-scans without contrast material of 2013 and 2014 revealed a newly appeared large (21 × 13 mm) nodule with a central calcification located in the left costophrenic angle and attached to the paravertebral chest wall (Fig. 2).

**Fig. 3:**
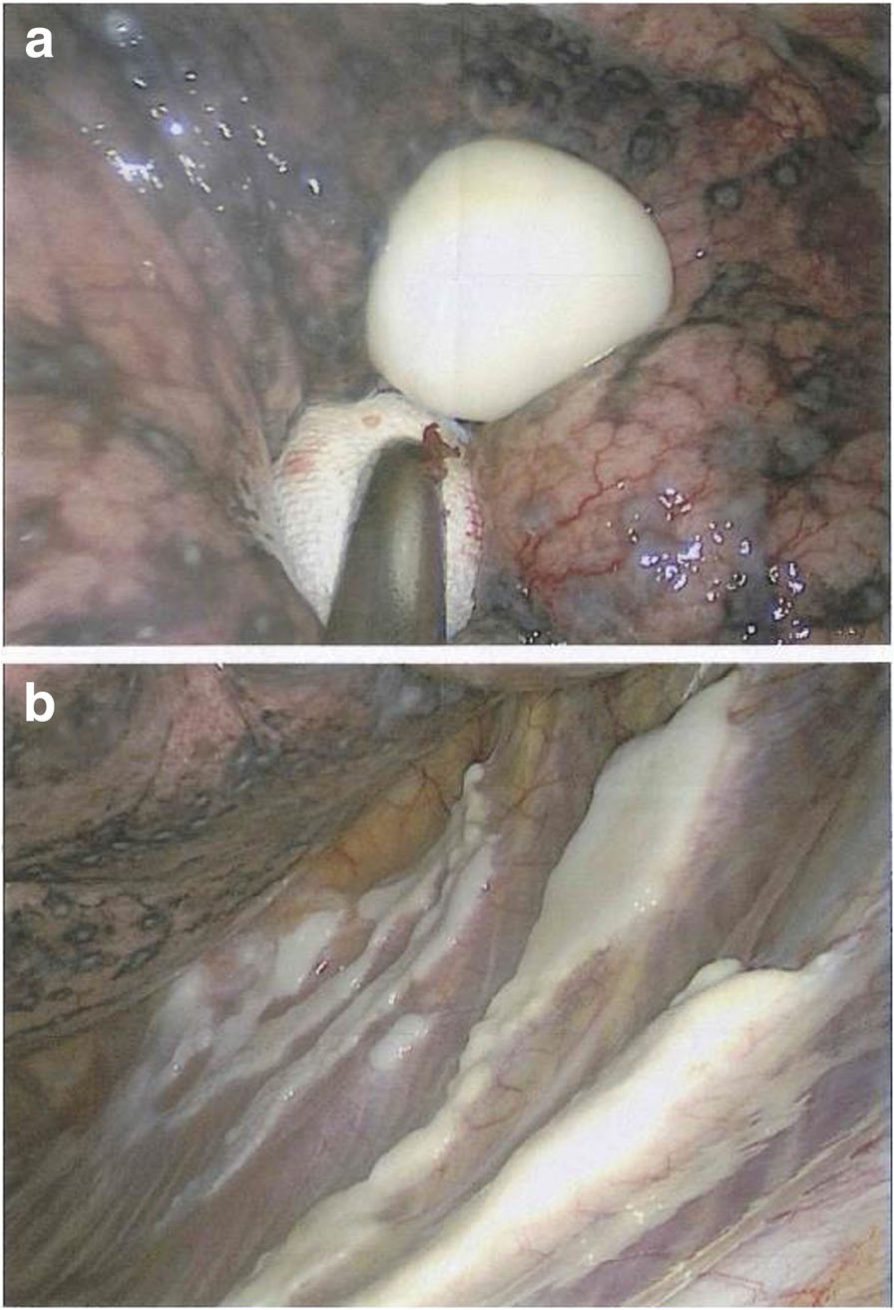
Situation of the parietal pleura close to the *left* costophrenic angle during operation with a spherical smooth mass (**a**) and multiple hyaline plaques (**b**)

**Fig. 4:**
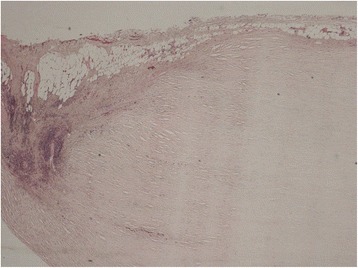
Histological image of the parietal pleura showing typical signs of a pleural plaque (fat tissue in the border area)

Based on the tentative diagnosis of a fast growing tumour with no radiological signs of malignancy and the urgent need of further diagnostic clarification, a left sided antero-axillary thoracotomy with atypical wedge resection of segment S10, removal of lymph nodes and ablation of the tumorous mass attached to the pleura was carried out. Figure 3 shows the situation of the parietal pleura close to the left costophrenic angle during the operation with ﻿a spherical smooth mass (3a) and multiple hyaline plaques located in the same area (3b). The nodule could be easily removed from the diaphragmatic pleura. After histological analysis the removed tissue was described as a hyaline pleural plaque with central calcification and no signs of asbestos bodies or malignancy (Fig. 4). The postoperative rehabilitation of the patient was uneventful. One year later lung function testing showed normal results for FEV1 and VC max. The described nodule was no longer visible on CT-scan. At that time the patient felt a slight discomfort in the left chest wall when taking a deep breath.

**Fig. 5:**
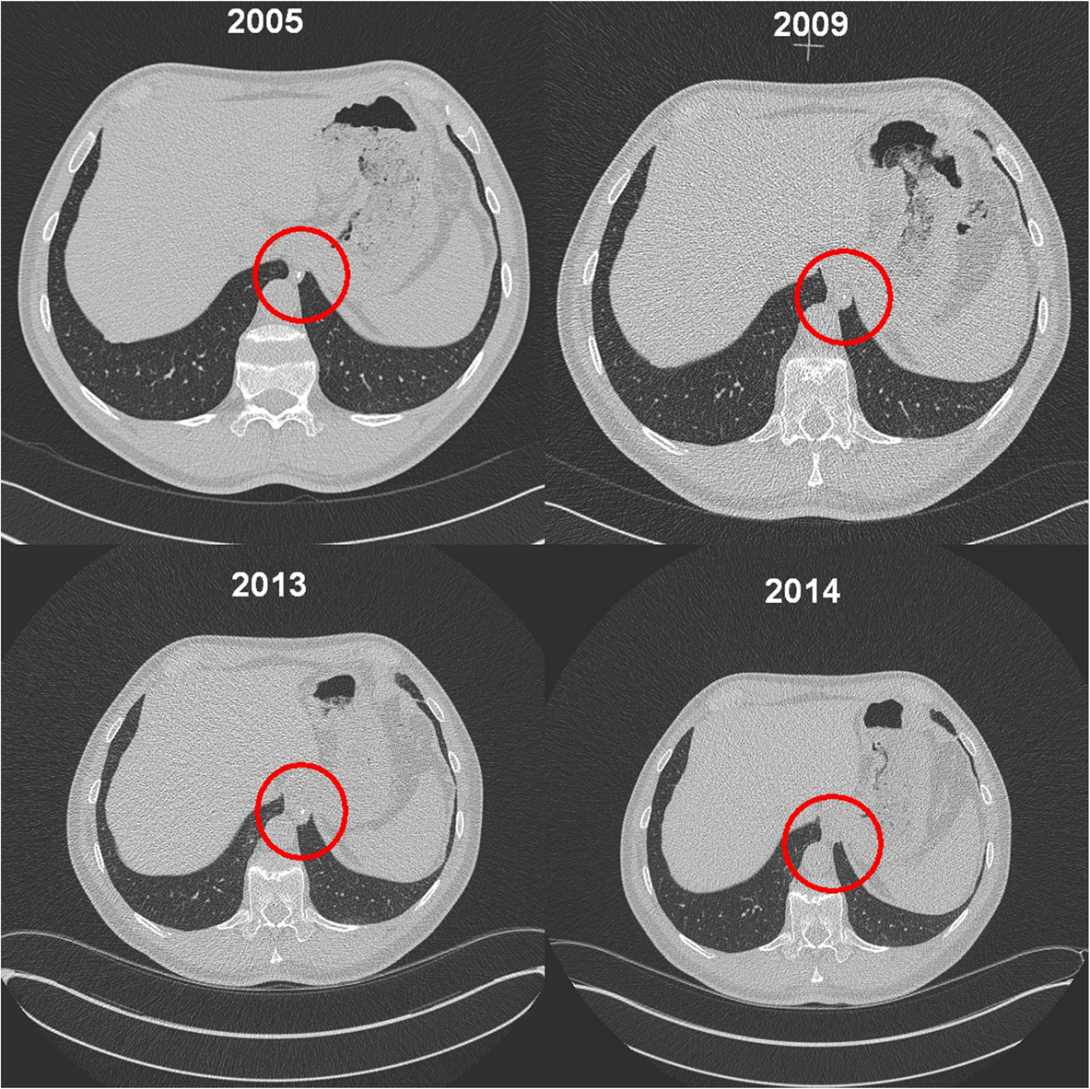
CT-scans taken between 2005 and 2013 showing the growing nodule attached to the mediastinal pleura next to the oesophageal hiatus. The discovery of the presumably same nodule further down the sinus in 2014 triggered further diagnostic interventions

**Fig. 6:**
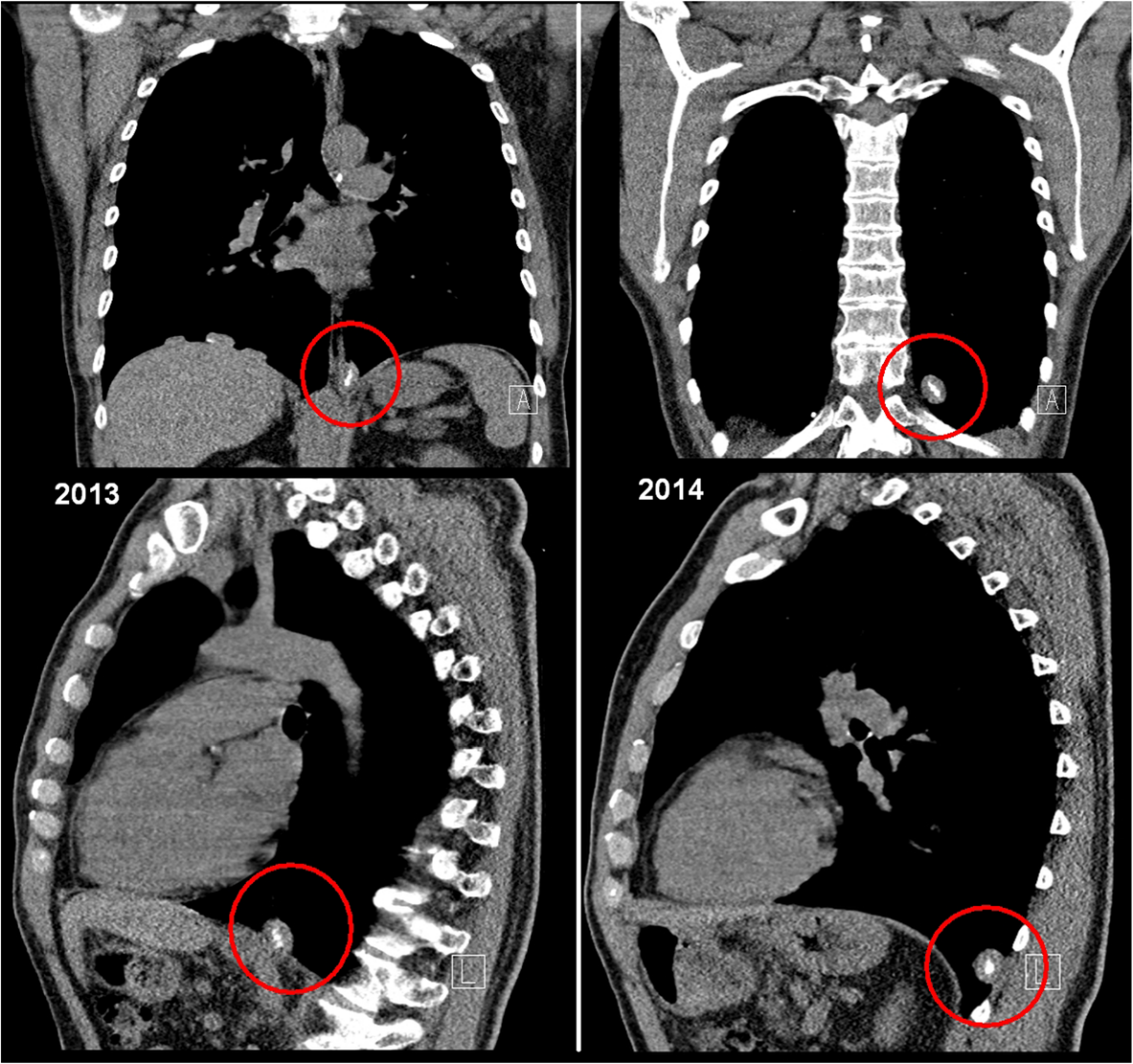
Location of a partly calcified nodule shown in lateral projection and comparing the situation in the years 2013 (*left*) and 2014 (*right*). It can be assumed that the dislocation of the nodule, later identified as a pleural plaque, took place sometime between the two scans. The dislocation was probably caused by physical exertions during a mountain hike and marked by an episode of thoracic pain shortly after the hike

An extensive review of all available previous CT-scans of the patient going back to the year 2005 revealed a rounded opacity with central calcification which had been attached to the mediastinal pleura close to the oesophagus and slightly cranial to the position of the removed nodule (Fig. 5). That opacity started as a small nodule hardly discernible from the surrounding tissue and slowly increased in size until the year 2014 when it emerged lower down in the costophrenic sinus and was described for the first time (Figs. 5 and 6). The other asbestos related changes of the chest wall and the diaphragmatic pleura showed no progress during the observation period.

The re-examination of the tissue in a second pathological laboratory confirmed the original result describing the nodule as a typical asbestos associated pleural plaque with the special feature of a pedunculated fixation point to the pleura. This was consistent with the observation that the plaque was not firmly adherent, which is atypical for plaques on the diaphragm [9].

The patient recalled participating in an extensive mountain hike over several weeks involving great physical exertions, which took place few weeks before the medical examination in the year 2014 when the nodule attached to the pleura was discovered. About 2 weeks after returning from the hike the patient developed massive pain in the thorax with dyspnoea at rest, which lasted for several hours. Other symptoms were not observed and he did not see a doctor at that time.

## Discussion and Conclusions

In this case of a formerly asbestos exposed patient with massive asbestos related changes of the pleura the clinical work up was complicated by a combination of two rather unusual features: the inconspicuous original location of the plaque and its detachment and migration within the pleural cavity, which lead to the tentative diagnosis of fast growing tumour. Despite repeated medical examinations over several years, the plaque developed unnoticed in an unusual location and continued to increase in size. The steady growth which could not be observed in any of the other plaques may be partly explained by its location on the diaphragm next to the oesophageal hiatus. One could speculate that the extreme motility in this area created an additional stimulus for growth or that the proximity to the oesophagus may have led to the accumulation of swallowed fibres creating a “hot spot” of plaque development. Alternative mechanisms such as the absorption of a foreign body through the oesophageal wall and its subsequent wandering or some kind of tumorous growth have been effectively ruled out by the results of the histological analysis [9–11].

When reviewing the series of CT-scans taken over a period of 10 years, it seems evident that the discovered nodule, after a phase of steady growth unnoticed by the CT-readers, had somehow migrated to the spot where it was eventually discovered. Although its location, structure, size and partial calcification, as well as the lack of any related symptoms, did not support the tentative diagnosis of fast growing tumour, it was difficult to identify the nodule as a dislocated pleural plaque at that time without taking the results of the previous CT-scans into consideration. We found no report in the literature indicating the possibility of migrating plaques or a hypothesis of the mechanism involved. For our case we propose the explanation that the physical exertion during a mountain hike somehow started the separation of the plaque. The wandering along the pleural cavity was probably facilitated by the continuous motions of diaphragm and lung combined with the effect of gravity.

It is important to note that even with the early discovery of the plaque at its original location, the operation with removal of the nodule and its careful examination would have been unavoidable. As the diagnosis of “migrating plaque” was unlikely, a CT-guided biopsy was considered too risky and tumorous growth could not be ruled out, the operation would still have been the recommended strategy.

The correct identification of pleural plaques is complicated by their similarity with other structures often leading to over-diagnosis, but they can also be overlooked through extended periods of time when developing in unusual locations. The possibility of migrating plaques mimicking fast growing tumours should be taken into consideration, particularly in patients with known asbestos related pleural changes.

## Abbreviations

CT: Computed tomography
FEV1: Forced expiratory volume in 1 s
VC max: Maximum vital capacity
